# Influence of Psychological Vulnerability Factors for Bipolar Disorders on a Semantic Mediated Priming Task

**DOI:** 10.3389/fpsyg.2021.598114

**Published:** 2021-06-09

**Authors:** Mélanie Labalestra, Nicolas Stefaniak, Laurent Lefebvre, Chrystel Besche-Richard

**Affiliations:** ^1^Service de Psychologie Cognitive et Neuropsychologie, Université de Mons, Mons, Belgique; ^2^Laboratoire Cognition Santé Société, Université de Reims Champagne-Ardenne, Reims, France

**Keywords:** bipolar disorder, vulnerability, affective temperaments, spreading activation, semantic memory

## Abstract

Hypomanic personality, hyperthymic temperament and irritable temperament are considered as psychological vulnerability factors to bipolar disorders. Semantic memory is impaired in bipolar patients. Spreading activation is among the probable candidates for accounting this impairment. The aim of this study was to assess spreading activation according to vulnerability factors continuum to determine whether it could be a factor of vulnerability to bipolar disorders. A sample of 61 healthy volunteers was recruited. Spreading activation was assessed by semantic mediated priming implemented in a double lexical decision task. Results shown that semantic mediated priming was negatively associated to hyperthymic temperament and irritable temperament. Impairment in semantic memory, and more specifically spreading activation, appear to be a cognitive factor of vulnerability to bipolar disorders. Our results can contribute to a better understanding of semantic impairment in vulnerable population and in bipolar disorder.

## Introduction

Bipolar disorders (BD) are characterized by a succession of manic (or hypomanic) and depressive episodes with remission periods between symptomatic ones. Cognitive dysfunctions in BD have been shown to affect verbal memory (Jabben et al., 2010), attention (Arts et al., 2007), executive functions (Bora and Pantelis, 2015), social cognition (Bora et al., 2016; De Siqueira Rotenberg et al., 2020), and verbal skills (Bortolato et al., 2015). Several studies have found deficits affecting verbal association, semantic contents, prosody and verbal fluency (Andreou et al., 2013; Radanovic et al., 2013). One hypothesis to explain these language-related issues is the impairment of spreading activation in semantic memory (Andreou et al., 2013), which refers to the fact that, when a concept is activated in memory, some of this activation automatically spreads to related concepts in semantic memory (Collins and Loftus, 1975). Automatic spreading activation is one of the processes that have been proposed to account for semantic priming (SP). SP corresponds to the fact that a target word (e.g., *cat*) is processed faster and more accurately when it is preceded by a semantically related word (e.g., *dog*) than when it is preceded by an unrelated word (e.g., *chair*).

The SP effect has already been investigated in BD, and the priming effect was reduced in comparison to control participants (Andreou et al., 2013). Although the results were interpreted as indicating an automatic spreading activation impairment, this interpretation could be challenged. Due to some methodological choices in Andreou et al.’s (2013) study, it is not certain that automatic spreading activation is the only process impaired in BD. Indeed, in Andreou et al.’s (2013) study, the SOA was 250 ms. Thus, in Cañas (1990), we cannot exclude the possibility that Andreou et al.’s results might be explained by an impairment of expectancy generation.

To cope with these limitations, some authors have used a mediated priming paradigm in which the semantic relationship is not direct but requires a mediating word (Chwilla and Kolk, 2002; Stefaniak et al., 2010). For instance, for the pair *lion* and *stripes*, there is no direct association between these words, but both are semantically related to *tiger*, which is the mediating word. The SOA used in this task is 0 ms that limits the involvement of strategic processes. By adopting this approach, we could determine more accurately whether spreading activation is impaired in people on the BD spectrum, and explain some of the related semantic and language problems.

In addition, three aspects of Andreou et al.’s (2013) samples should be noted. First, sample sizes for both patients and control participants were small (i.e., 14 outpatients and 12 controls); second, the sex ratio was not respected in their study since there only two males, while the prevalence is quite similar in both sexes; third, although their patients were euthymic, they were undergoing medical treatment to ensure their stabilization, and these drugs could have had a negative impact on their cognitive abilities (Delaloye et al., 2009). One way to circumvent the impact of this treatment is to recruit non-clinical individuals with psychological vulnerability factors for BD. Indeed, the heterogeneous expression of BD symptoms (severity, forms) has led some authors to propose that there is a bipolar spectrum (Akiskal and Mallya, 1987). This dimensional approach views BD as a continuum ranging from non-pathological to pathological forms. On the non-pathological side, there are various personality profiles (e.g., hypomanic personality) and affective temperaments (e.g., hyperthymic and irritable temperaments) that are considered to be psychological vulnerability factors for BD (Pompili et al., 2018). In other words, these personality profiles and affective temperaments are subclinical phenotypical manifestations of BD (Akiskal and Akiskal, 2005; Bentall et al., 2011; Perugi et al., 2012). The characteristics of each affective temperaments are presented in Table 1.

**TABLE 1:** Description of affective temperaments (Akiskal and Akiskal, 2005).

Affective temperaments	Description
Cyclothymic	Unstable mood and energy fluctuations (motor and cognitive abilities).
Depressive	Sensitive, with a lack of energy, putting the interests of others before their own, tendency to see only the negative side of things.
Hyperthymic	Exuberant, gregarious, talkative, cheerful, expansive.
Irritable	Explosive for no apparent reason, brawling, impulsive, may display pathological drunkenness.
Anxious	Fearful, afraid to learn bad news about loved ones.

To the best of our knowledge, only two studies had explored semantic processes in a population vulnerable to BD. Both studies found abnormalities affecting semantic inhibition processing (Raucher-Chéné et al., 2017, 2018), that is controlled processes. Thus, semantic memory for controlled processes seems to be impaired in hypomanic individuals, but nothing is known about automatic semantic spreading activation in vulnerable populations. Moreover, only one side (hypomanic personality) was explored in these studies, whereas affective temperaments were not.

Our aim is to explore how individual variability in psychological vulnerability factors (hypomanic personality and affective temperaments) affects automatic spreading activation. Andreou et al. (2013) found reduced priming in BD patients in a euthymic state. Although there are methodological differences, we also expected a reduced priming effect for individuals with higher levels of vulnerability factors to BD.

## Materials and Methods

Sixty-one healthy individuals (35 women and 26 men) took part in the study. They were students and staff members at the University of Reims Champagne-Ardenne (France) (*n* = 30) and University of Mons (Belgium), and they are between 18 and 60 years old. All participants were French native speakers and had normal or corrected-to-normal vision, normal hearing, and no reported past or present history of reading disabilities. Based on assessment with the Mini International Neuropsychiatric Interview (MINI, French version 5.0) (Lecrubier et al., 1997), no participant reported past or present neurological or psychiatric disorders. Moreover, none of them were taking any psychotropic drugs. A brief neuropsychological assessment suggested that the participants did not have any cognitive impairments that could interfere with experimental tasks (verbal fluency test and Stroop test of GREFEX battery, Godefroy and GREFEX, 2012; the Hayling test, Bayard et al., 2017; and the direct and reverse span, Wechsler, 2011). Verbal IQ was assessed with the Mill Hill Vocabulary Test (Deltour, 1998). Subclinical characteristics were assessed with the short French version of the Temperament Evaluation of the Memphis, Pisa, Paris, and San Diego Autoquestionnaire (TEMPS-A) (Krebs et al., 2006), the French version of the Hypomanic Personality Scale (HPS) (Terrien et al., 2015) and the Beck Depression Inventory (BDI) (Beck et al., 1961).

Sociodemographic data, scores on the different personality and temperament scales, and neuropsychological assessment are presented in Table 2.

**TABLE 2:** Mean and standard deviation (SD) for sociodemographic data, different personality and temperament scales and neuropsychological assessment (*N* = 61).

	*Mean*	*SD*
Age (years)	30.49	12.41
Education (years)	14.80	2.55
Mill-Hill (estimated IQ)	103.52	11.08
BDI	4.57	3.71
HPS Total	15.95	4.94
HPS Excitation	1.39	0.82
HPS Hypomania	5.85	2.97
HPS Social vitality	8.69	1.03
TEMPS-A Hyperthymic	3.77	1.82
TEMPS-A Irritable	1.03	1.45
TEMPS-A Depressive	1.82	1.30
TEMPS-A Cyclothymic	2.59	1.94
TEMPS-A Anxious	0.89	0.82
Phonological fluency (total number)	27.52	5.02
Semantic fluency (total number)	35.38	5.99
Direct span (WAIS-IV subscore)	10.85	1.91
Reverse span (WAIS-IV subscore)	9.61	1.61
‘Stroop naming (seconds)	54.20	7.23
Stroop naming (errors)	0.16	0.42
Stroop reading (seconds)	40.10	5.69
Stroop reading (errors)	0.10	0.35
Stroop interference (seconds)	88.13	14.18
Stroop interference (errors)	1.61	1.56
Hayling task part A (seconds)	1.39	0.82
Hayling task part A (errors)	0.08	0.33
Hayling task part B (seconds)	4.84	2.10
Hayling task part B (errors)	2.21	1.25

*BDI, Beck Depression Inventory; HPS, Hypomanic Personality Scale; TEMPS-A, Temperament Evaluation of the Memphis, Pisa, Paris, and San Diego Autoquestionnaire; WAIS-IV, Wechsler Adult Intelligence Scale, 4th edition.*

This research was approved by the local ethics committee at the University of Mons. The study was designed in accordance with the Declaration of Helsinki. All participants gave their written informed consent before inclusion in the study.

The semantic mediated priming task used in the study is a revised version of the one used by Stefaniak et al. (2010). The stimuli consisted of 356 prime-target word pairs presented at the center of a computer screen. A total of 712 letter strings were used: 532 French words and 180 pseudowords. The pseudowords were built according to the phonotactic constraints of the French language (for details of stimulus selection, see Stefaniak et al., 2010). Two versions of the task were constructed. Each version contained 29 related pairs, 29 counterbalanced pairs (control pairs), 30 unrelated filler pairs and 90 pairs with a pseudoword. For the pairs with a pseudoword, the pseudoword occurred as often in the prime position as in the target position. The counterbalanced pairs were built by rearranging the related pairs in one version to become unrelated pairs in the other version. The unrelated pairs (counterbalanced and filler pairs) were matched for frequency and length. The proportion of related pairs was 0.163.

To create related pairs, 200 triplets (i.e., prime – mediator – target) were generated *a priori*. In order to determine the semantic distance between the prime and the target for each triplet, a total of 100 undergraduate students – not involved in the experimental portion of the research – were asked to perform a free association task. Half of the students were asked to list the first five words that came to mind when thinking of the prime word. The other half were asked to perform the same free association task on the mediating word from the triplets. This dissociation aimed to prevent responses to the mediators from being influenced by the prime words. Triplets were chosen based on selection criteria inspired by Anaki and Henik (2003): the mediator should have a mean frequency superior to.20 or should be the word the most often given in response to the prime in the free association task. The target had to meet the same criteria in response to the mediator and should not be produced in response to the prime word. Of the 200 initial triplets, 58 met the selection criteria and were equally distributed between the two versions of the task (i.e., 29 items in each version of the task).

The computer used for the study was a Sony PCG-7186M and the priming task was programmed on OpenSesame software, version 2.9.7 (Mathôt et al., 2012). Each experimental trial began with a fixation cross displayed for 500 ms in the middle of the computer screen. The fixation cross was followed by the prime and the target stimulus (i.e., two letter strings), which were presented together for 400 ms on the left and right sides of the screen. The simultaneous presentation of the prime and the target meant that the SOA was equal to zero. After the presentation of the letter strings, a blank screen appeared and participants were asked to decide as fast and as accurately as possible whether both letter strings were French words or not. Stickers with “yes” and “no” were pasted on the M and Q keys, respectively, of an AZERTY keyboard. Response keys were reversed for left-handed participants so they could respond “yes” with their dominant hand. Participants had to press the “yes” key if both letter strings were French words and “no” if one of the two letter strings was not a French word. The proportion of expected “yes” responses was.51. The stimuli were randomly presented. The experimental session was preceded by five practice items. Halfway through the experimental session, participants were given a break. Participants were randomly assigned to one of the two versions.

## Results

In order to determine whether SP occurred, we performed an ANOVA to compare mean reaction times (RTs) of correct responses for Type of pair (4 conditions: related, counterbalanced, filler, pseudoword). Contrast analyses were conducted to determine whether the related pairs were processed faster than the counterbalanced ones.

To investigate the link between the level of vulnerability factors for BD and the priming effect, we performed both a null hypothesis test and Bayes Factor (BF) correlation analyses between the mediated priming effect and the scores for the different scales used to assess vulnerability factors. BF results are interpreted as follows: BF > 3 suggests the presence of a correlation; BF < 0.33 suggests the absence of a correlation; and 0.33 < BF < 3 is considered to lack sensitivity (Dienes, 2011). The value of BF is that they are relatively insensitive to multiplication of type I errors. BF were obtained using the BayesFactor R package (Morey et al., 2015).

The analysis of variance revealed a main effect of Type of pair, *F*(3,125) = 134.15, *p* < 0.001, η*^2^_*p*_* = 0.69, *MSE* = 6897.83. Contrast analyses showed that RTs were shorter for mediated related pairs than for counterbalanced ones, meaning that SP occurred. This was confirmed by the BF > 1000. The other conditions were also processed more slowly than the related pairs. These analyses are summarized in Table 3.

**TABLE 3:** Mean (SD) RTs for each of type of pair and contrast analysis (and BF) comparing each type to the semantically related pairs.

	Mean	SD	Mean difference from related pairs	*t*	*p*	BF
Related pairs	459.00	131.34	–		–	
Counterbalanced pairs	501.30	40.48	−42.30	7.71	<0.001	>1000
Filler pairs	592.97	184.83	−133.97	8.69	<0.001	>1000
Pseudoword pairs	683.62	209.29	−278.22	15.65	<0.001	>1000

The correlation analyses (Table 4) revealed significant negative correlations between the mediated priming effect and the social vitality subscale of the HPS, and the irritable and hyperthymic temperaments of the TEMPS-A. These results remained significant when age, education and verbal IQ (Mill-Hill score) were controlled in the analysis (social vitality: *r* = –0.27, *p* = 0.04; irritable: –0.39, *p* = 0.003; hyperthymic: *r* = –0.47, *p* < 0.001). However, when Holm corrections were applied, the association between social vitality and the priming effect was no longer significant (*p* = 0.29). This absence of association is confirmed by the BF < 3.

**TABLE 4:** Correlation analysis between mediated priming effect and vulnerability factors for BD.

	Pearson *r*	*p*	Adjusted *p* (Holm)	BF
BDI total	−0.10	0.46	1	0.33
HPS total	−0.27	0.04	0.29	1.70
HPS Social vitality	−0.27	0.04	0.29	1.74
HPS Hypomania	−0.21	0.11	0.67	0.77
HPS Excitation	−0.16	0.22	1	0.50
TEMPS-A Cyclothymic	−0.10	0.46	1	0.33
TEMPS-A Depressive	−0.03	0.85	1	0.26
TEMPS-A Irritable	−0.39	0.002	0.02	19.20
TEMPS-A Hyperthymic	−0.48	< 0.001	0.001	226.80
TEMPS-A Anxious	−0.10	0.44	1	0.34

## Discussion

We showed that a mediated priming effect occurred, which makes it possible to interpret our results in terms of spreading activation. Indeed, semantic mediated priming tasks are considered especially relevant for measuring spreading activation in semantic memory since, given that semantic activation from the prime must spread beyond the mediator to activate the target word, strategic processes are unlikely to be involved in the SP effect.

Our results showed negative associations between the mediated priming effect and two affective temperaments: irritable and hyperthymic. Thus, spreading activation seems to function less efficiently in individuals with higher levels of vulnerability factors for BD.

Previous studies showed that controlled processes are impaired in a population vulnerable to BD (Raucher-Chéné et al., 2017, 2018). Our study shows that, in addition to an alteration in these controlled processes, the automatic process of spreading activation is also less efficient. The impairment of both automatic and controlled processes in semantic memory could make it difficult for the vulnerable population to use compensation strategies for semantic processing. If both automatic and controlled processes are impaired in semantic memory, that could lead to difficulties processing contextual information or understanding a situation (automatic process deficit), along with an inappropriate selection of information and/or hyperactivation of too much information at the same time (controlled process deficit). Taken together, these language issues can be observed in subclinical manic symptomatology such as hyperthymic and irritable temperaments. In fact, these temperaments are associated with impulsivity or exuberant behavior, which could be the result of a lack of inhibition. However, the characteristics of individuals who present a controlled process deficit seem to be different from those of people with a deficit affecting automatic processes. In previous studies, controlled process deficits were found for the vitality facet of hypomanic personality, which corresponds to social potency and vivaciousness. In our study, only irritable and hyperthymic temperaments were associated with disrupted automatic processes. These temperaments correspond to exuberant or inappropriate behaviors. Thus, these results suggest that the consequences of different semantic impairments are revealed in the expression of different symptoms.

In our study, hypomanic personality and affective temperaments were assessed by self-report, which can lead to over- or underestimation of symptoms. However, previous studies showed that the HPS and TEMPS-A provided a good assessment of the quantitative and qualitative extent of symptoms (Kwapil et al., 2000; Akiskal et al., 2005). In other respects, our study is the first to show disruption of automatic semantic processes in hyperthymic and irritable populations. Future studies are needed to replicate the results. Furthermore, studies are also needed concerning other cognitive functions in these populations, for which few studies exist to date. It could also be interesting to carry out a comparative study between the irritable and hyperthymic temperaments to identify the processes’ impact on each other.

In conclusion, this study shows reduced automatic spreading activation in individuals with higher levels on scales that measure vulnerability factors for BD. Characteristics of these psychological vulnerability factors are present during the manic state of BD, suggesting that semantic abnormalities could be manic-specific. Indeed, by investigating the continuum of psychological vulnerability factors, our results suggest that impaired spreading activation process may be a cognitive vulnerability factor for BD. Thus, if first-degree relatives had altered spreading activation, this could corroborate the results of our study. Furthermore, due to the impact of language problems on daily functioning, it is important to clarify the nature and extent of the semantic dysfunctions in BD, and more generally, in the bipolar spectrum.

Our study has limitations. For example, it does not take into account the role of genetic and environmental risk factors in the assessment of vulnerability to BD. For example, child maltreatment or child abuse can be considered as risk factors for negative outcomes (Serafini et al., 2017) and are known for affecting memory processes. In addition, we know the sensitivity of bipolar in the processing and regulation of emotional information (Lima et al., 2018). Sensory processing for emotional information or other personality traits as alexithymia (Serafini et al., 2016) involved in the quality of life of bipolar patients could be explored in the future in relation to semantic processes.

Our study helps to show that the impairments of semantic memory in BD are not due to the condition *per se* but, on the contrary, constitute a factor that might increase the conversion rate to BD in individuals when it is more impaired. Developing ecological, rapid and easily usable methods allowing the evaluation of semantic processes constitutes the future challenge to be taken up in order to improve the screening tools for the risk of psychopathological transition, particularly toward bipolar disorders.

## Data Availability Statement

The raw data supporting the conclusions of this article will be made available by the authors, without undue reservation.

## Ethics Statement

The studies involving human participants were reviewed and approved by the local ethical committee (University of Mons). The patients/participants provided their written informed consent to participate in this study.

## Author Contributions

CB-R, LL, and NS designed the project, contributed to the conception and the development of the manuscript and reviewed its several drafts. ML conducted the study, collected and wrote the drafts, and the final manuscript. ML and NS analyzed the data. All authors approved it for publication.

## Conflict of Interest

The authors declare that the research was conducted in the absence of any commercial or financial relationships that could be construed as a potential conflict of interest.
